# Microbial population analysis of the midgut of *Melophagus ovinus via* high-throughput sequencing

**DOI:** 10.1186/s13071-017-2323-1

**Published:** 2017-08-09

**Authors:** De-Yong Duan, Guo-Hua Liu, Tian-Yin Cheng, Ya-Qin Wang

**Affiliations:** 1 College of Veterinary Medicine, Hunan Agricultural University, Changsha, Hunan Province 410128 China; 2 Hunan Co-Innovation Center of Animal Production Safety, Changsha, Hunan Province 410128 China

**Keywords:** Microbial community structure, Midgut, *Melophagus ovinus*, Illumina HiSeq, 16S rDNA

## Abstract

**Background:**

*Melophagus ovinus*, one of the most common haematophagous ectoparasites of sheep, can cause anaemia and reductions in weight gain, wool growth and hide value. However, no information is available about the microfloral structure of the midgut of this ectoparasite. In the present study, we investigated the microbial community structure of the midgut contents of fully engorged female and male *M. ovinus* using Illumina HiSeq.

**Results:**

The phylum showing the highest abundance was Proteobacteria (99.9%). The dominant bacterial genera in females and males were *Bartonella*, *Arsenophonus* and *Wolbachia*. Some less abundant bacterial genera were also detected, including *Enterobacter*, *Acinetobacter*, *Halomonas*, *Shewanella*, *Bacillus* and *Staphylococcus*.

**Conclusions:**

*Bartonella*, *Arsenophonus* and *Wolbachia* were the dominant bacterial genera in the midgut of female and male *M. ovinus*. Although detected, *Enterobacter*, *Acinetobacter*, *Halomonas*, *Shewanella*, *Bacillus* and *Staphylococcus* showed low abundances. Importantly, this is the first report of the presence of *Arsenophonus*, *Wolbachia*, *Enterobacter*, *Halomonas*, *Shewanella*, *Bacillus* and *Staphylococcus* in the midgut of *M. ovinus*.

## Background

*Melophagus ovinus*, also termed the sheep ked, is a wingless fly that belongs to the order Diptera, family Hippoboscidae. *Melophagus ovinus* is one of the most common haematophagous ectoparasites of sheep [1] and is mainly found on the animal’s neck, shoulder and perineal regions and between the hind legs. The life-cycle of *M. ovinus* includes four developmental stages: larva, pupa, nymph and adult, all of which occur in the wool of the host [2]. Although sheep are generally considered to be a definitive host of this ectoparasite, *M. ovinus* can also parasitize the body surfaces of goats [3], European bison [4], rabbits and humans [5] and red foxes [6]. The number of *M. ovinus* individuals within a flock varies significantly over the annual cycle, increasing on ewes throughout winter until the lambing season in March and *M. ovinus* and decreasing on adults while rapidly increasing on lambs until a peak in May; the flock’s total population reaches a minimum from April-May [7].

The blood-feeding process of *M. ovinus* causes harm to the host in two ways. First, *M. ovinus* can cause pruritus and inflammation; as the host attempts to rub, scratch and bite the parasitized location, some wool will be lost, and the skin will also be damaged, leading to secondary microbial infections and establishing conditions for cutaneous myiasis [2, 7–10]. A large number of *M. ovinus* parasitizing a single sheep can result in anaemia and reductions in weight gain and wool growth [11] as well as hide value [2, 7, 12].

Secondly, as a vector, *M. ovinus* can transmit *Trypanosoma melophagium* [12], *Anaplasma ovis* [13], *Acinetobacter* [2, 13] and *Borrelia burgdorferi* [14]. In addition, Luedke et al. [15] reported that *M. ovinus* can mechanically transmit blue-tongue virus, which causes a serious infectious disease in sheep. Recently, *Rickettsia* was also detected at a high prevalence (12.63%, 12/95) in *M. ovinus* from Taklimakan Desert in China, and phylogenetic analysis confirmed the presence of *R*. *raoultii* and *R*. *slovaca* [8].

Some studies have reported negative results for vector-borne pathogens via molecular screening of *M. ovinus*. For example, Hubálek et al. [16] found that tick-borne encephalitis virus and other arboviruses were not present in *M. ovinus*. Similarly, Nelder et al. [17] screened a 150 *M. ovinus* individuals for *Coxiella burnetii*, with negative results. Rudolf et al. [18] also failed to find evidence of flaviviruses, phleboviruses, bunyaviruses, *Borrelia burgdorferi*, *Rickettsia* spp., *Anaplasma phagocytophilum* or *Babesia* spp. in *M. ovinus*.

However, previous studies on *M. ovinus*, including those with positive or negative results, have focused on one specific pathogen, whereas a comprehensive study on bacteria associated with *M. ovinus* (including pathogenic and symbiotic bacteria) has yet to be conducted.

Illumina HiSeq is an expedient and efficient method for analysing microbial community structure, with the following advantages: (i) bacterial identification does not depend on bacterial culture, and (ii) these techniques can identify bacterial species present at low relative abundance, providing more precise microbial population information [19, 20]. Investigation of the 16S rDNA region can be used for species identification and as an index for microbial systematics, classification and identification. The V3-V4 hypervariable regions are the most accurate and can identify organisms at the genus level [21].

The aim of this study was to apply Illumina HiSeq based on the V3-V4 hypervariable regions of the 16S rDNA region to examine the microbial community structure of the midgut of *M. ovinus* to determine which pathogenic and symbiotic bacteria were carried by *M. ovinus*. The findings may lead to a strategy for preventing infestations of *M. ovinus* and vector-borne pathogens.

## Methods

### Sample collection and DNA extraction

All of the *M. ovinus* individuals used in this study were obtained from sheep bred in the city of Jiuquan in Gansu Province (1500 m above sea level; 39°71′N, 98°50′E), China, in October 2016. The samples of *M. ovinus* were removed using forceps from wool at the neck, shoulder and perineal region and between the hind legs (the sheep were all bred by the same farmer and had same environment). The *M. ovinus* samples were immediately transported to the Laboratory of Molecular Physiology of the College of Veterinary Medicine, Hunan Agricultural University. Five adult females (fully engorged) and 5 adult males (fully engorged) *M. ovinus* were examined in this study. All *M. ovinus* specimens were processed as individual samples. DNA extraction procedures were performed in a biosafety cabinet to ensure protection of the samples from environmental contamination. First, the *M. ovinus* samples were washed three times in 70% ethanol for 2 min, followed by a wash with sterile deionized water to remove environmental debris and to disinfect the surface. The *M. ovinus* individuals were stabilized with fine-tipped forceps by holding their rear portions; the forceps were inserted into the rear of each *M. ovinus,* and the dorsum was sliced to expose the organs. The midgut was removed, and the contents were extruded and suspended in 0.01 M phosphate-buffered saline (PBS, pH 7.3, including NaCl, KCl, KH_2_PO_4_ and Na_2_HPO_4_.12H_2_O). The contents were then centrifuged at 300 r/min for 5 min, and the supernatant was retained. A 1 ml aliquot of the supernatant was centrifuged at 10,000 r/min for 1 min, the precipitates were subjected to DNA extraction using a TIANamp Bacteria DNA kit (TianGen Biotech Corporation, Beijing, China) according to the manufacturer’s protocol. The DNA concentration was determined by 1% agarose gels electrophoresis. The DNA samples were diluted to a concentration of 1 ng/μl using sterile water and stored at -20 °C until analysis.

### Sequencing

#### Amplicon generation

The primers 341F (5′-CCT AYG GGR BGC ASC AG-3′) and 806R (5′-GGA CTA CNN GGG TAT CTA AT-3′) with sample-identifying barcodes were used to amplify the V3-V4 hypervariable regions of the bacterial 16S rDNA.

A polymerase chain reaction (PCR) mixture (20 μl per reaction) was prepared with 15 μl Phusion® High-Fidelity PCR Master Mix (New England BioLabs, Ipswich, USA), 0.2 μM forward and reverse primers, and 10 ng template DNA. The reaction was as follows: initial denaturation at 98 °C for 1 min, followed by 30 cycles of 98 °C for 10 s, 50 °C for 30 s and 72 °C for 60 s, and a final elongation at 72 °C for 5 min. A negative control (sterile water) was included with each reaction. The PCR products were stored at 4 °C overnight or were frozen until use.

#### PCR product quantification and qualification

The PCR products (with 5 μl of 1× gel loading buffer) were subjected to 2% agarose gel electrophoresis at 70 V for 50 min; each sample separated by an empty well. The bands were excised (the expected size was between 200 and 250 bp) using a clean scalpel, weighed, and extracted and purified using a GeneJET Gel Extraction Kit (Thermo Fisher Scientific, Waltham, USA). Each PCR product was quantified, and dilutions were performed to obtain a stock solution at 1 μg/ml.

#### Library preparation and sequencing

Sequencing libraries were generated using a TruSeq® DNA PCR-Free Sample Preparation Kit (Illumina, San Diago, USA) following the manufacturer’s protocol; index codes were added. The library quality was assessed using a Qubit @ 2.0 Fluorometer (Thermo Fisher Scientific) and quantitative PCR (Q-PCR). The libraries were sequenced on the Illumina HiSeq platform, and 250 bp paired-end reads were generated.

### Data analysis

Paired-end reads with unique barcodes were trimmed to remove the barcodes and primers. To obtain raw tags, the trimmed reads were assembled using the FLASH software package (Version 1.2.7) [22]. This program was used to merge the paired-end reads when at least some overlapped with the reads produced by the same DNA fragment’s opposite end. Raw tags were analysed using QIIME software (Version 1.7.0) [23] under specific filtering conditions to obtain high-quality clean tags (effective tags). Additionally, chimeric sequences were removed using the UCHIME algorithm (http://www.drive5.com/usearch/manual/uchime_algo.html), comparing the tags to Unite Reference Database (https://unite.ut.ee/) to detect chimeric sequences; effective tags were ultimately obtained. Uparse software (Version 7.0.1001) [24] was used to cluster the sequences with 97% similarity into operational taxonomic units (OTUs).

A representative sequence for each OTU was screened for further annotation. The taxonomy of the OTUs was obtained using QIIME software (Version 1.7.0) against Unite Database (https://unite.ut.ee/). MUSCLE software (Version 3.8.31) [25] was used to derive the phylogenetic relationships among OTUs via multiple sequence alignment. Alpha and beta diversities were calculated based on normalized OTU abundance information, which was obtained using the sample with the fewest sequences as a standard. Indices, including observed species, Shannon’s diversity index and Simpson’s diversity index and Good’s coverage, were calculated with QIIME (Version 1.7.0), and the results are displayed using R software (Version 2.15.3).

## Results

### Morphological characteristics of *Melophagus ovinus*

**Fig. 1:**
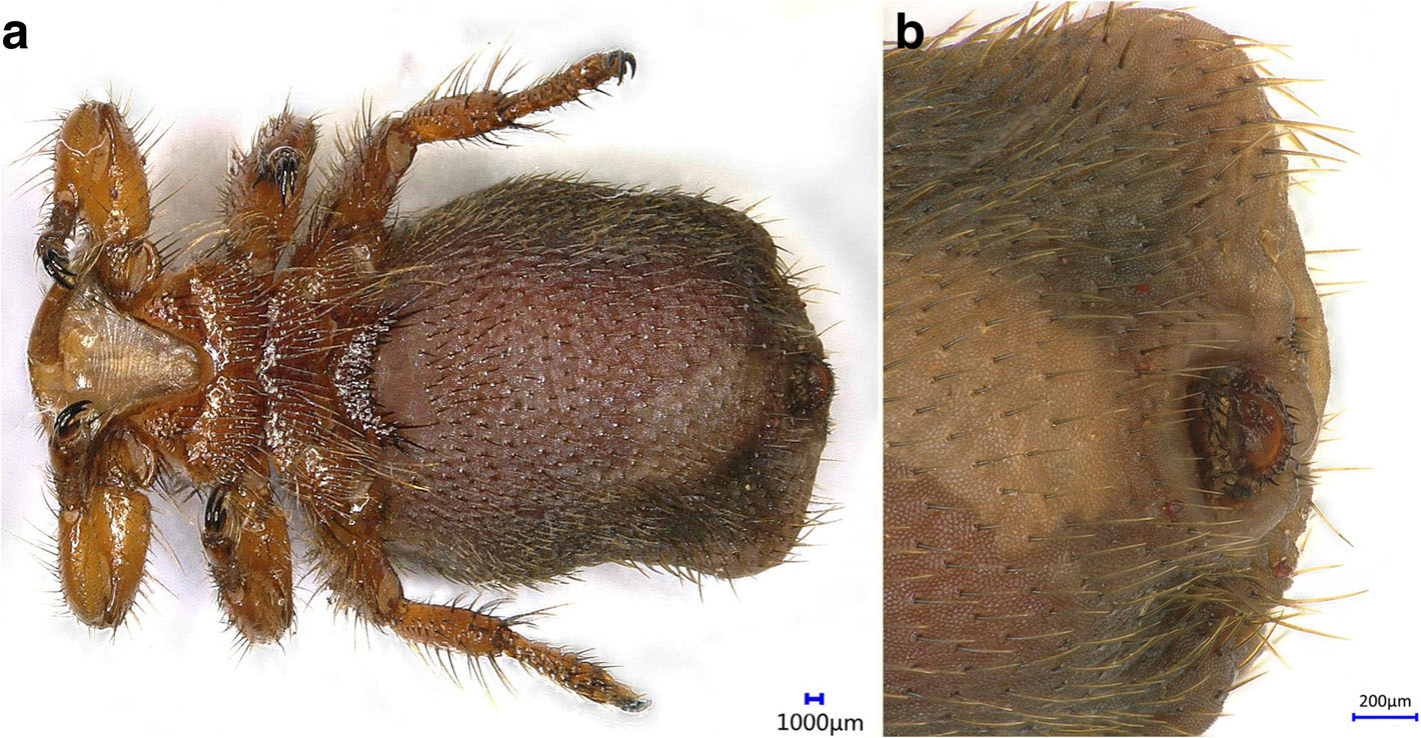
Photomicrographs of female *Melophagus ovinus*. **a** Ventral view of the female. **b** Posterior end of the female

**Fig. 2:**
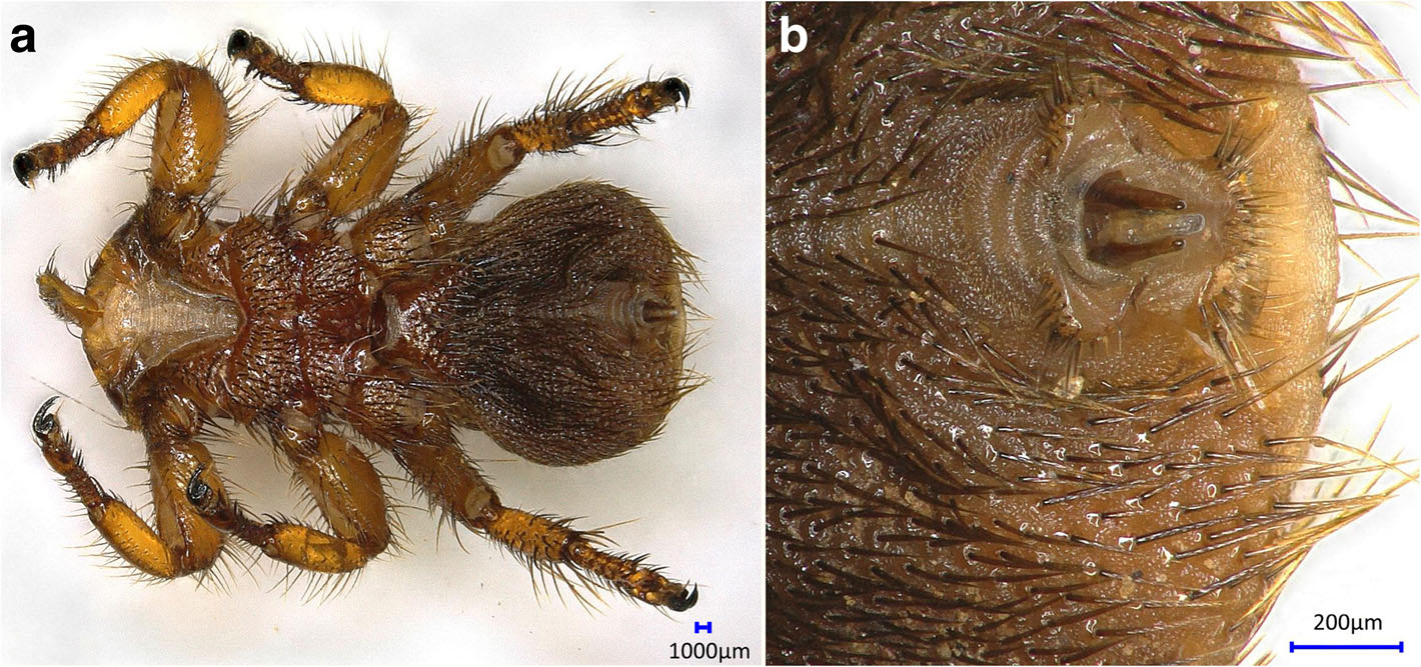
Photomicrographs of male *Melophagus ovinus*. **a** Ventral view of the male. **b** Posterior end of the male

*Melophagus ovinus* is an entirely wingless brown fly (Dipteran: Hippoboscidae). Both sexes have three pairs of legs, and the tibial ends have large claws that enable *M. ovinus* to maintain its position in the sheep’s fleece (Figs. 1a, 2a). The insect has piercing-sucking mouthparts, reduced compound eyes and antennae. Body length ranges from approximately 4.0 to 6.2 mm; the body wall is leathery, and the entire surface exhibits dense setae. The head is short and embedded in the chest; the abdomen is wide and ameristic, with an oval or round shape. The *M. ovinus* abdomen differs depending on the sex: the female’s abdomen is large, round, and invaginated on the back (Fig. 1b), whereas the male’s abdomen is small, round, and embossed on the back (Fig. 2b).

### General statistics

Sequencing of the amplicons of the V3-V4 hypervariable regions of the bacterial 16S rDNA produced a number of reads for the female and male *M. ovinus* samples evaluated. A total of 145,679 sequences were obtained after barcodes and primer sequences were trimmed, and following quality control, 124,707 high-quality, effective tags were generated for analysis using OTU selection and taxonomic assignments.

The alpha diversity indices of the bacterial communities of female and male *M. ovinus* are shown in Table 1. Shannon’s diversity index and Simpson’s diversity index were similar for the female and male samples, suggesting that fully engorged females and males have similar bacterial community distributions. Good’s coverage rates of all samples were 100%, indicating that the sequencing depths were sufficient to saturate the bacterial diversity, with the majority of bacteria in the two samples being previously described.

**Table 1:** Indices of bacterial abundance and diversity in samples

Sample	No. of species observed	Shannon’s index	Simpson’s index	Good’s coverage
Female	9	1.199	0.524	1
Male	8	1.098	0.495	1

The Shannon’s diversity index and Simpson’s diversity index were used to estimate the total number of species in the community. Observed species was the number of species observed in a sample. Good’s coverage index was used to estimate the percentage of total bacterial OTUs represented in a sample

**Fig. 3:**
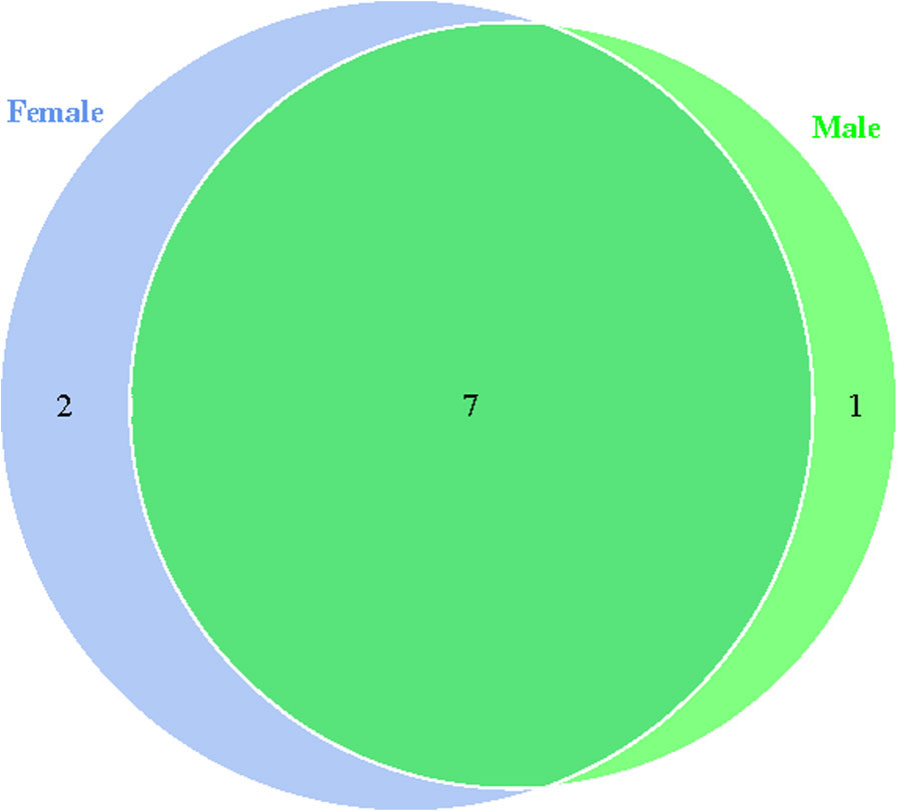
Venn diagrams of two midgut content samples based on OTUs

OTU cluster analysis is shown as a Venn diagram in Fig. 3. Nine and 8 OTUs were obtained for female and male *M. ovinus*, respectively. Seven of the OTUs showed high similarity between the two groups, indicating that fully engorged females and males have similar microbial populations.

In all samples, the majority of tags were classified, and more than 99% of tags were assigned to a genus. Only a small fraction of tags could be classified at the species level.

### Microbial population characteristics

Proteobacteria and Firmicutes were the main bacterial phyla in fully engorged female and male *M. ovinus*. Proteobacteria comprised 99.9% of the OTUs in the two groups, showing a marked predominance. The microbial population characteristics at the genus level are shown in Table 2. *Bartonella*, *Arsenophonus*, *Wolbachia*, *Enterobacter*, *Acinetobacter*, *Halomonas*, *Shewanella*, *Bacillus* and *Staphylococcus* were detected in all samples, with *Bartonella*, *Arsenophonus* and *Wolbachia* predominating. Although *Enterobacter*, *Acinetobacter*, *Halomonas*, *Shewanella*, *Bacillus* and *Staphylococcus* were detected, abundance for these taxa was low. *Arsenophonus* and *Wolbachia* were abundant in females (55.69 and 3.34%, respectively) but were less abundant in males (38.14 and 1.67%, respectively). In contrast, *Bartonella* was more abundant in males (59.91%) than in females (40.56%), though the number of samples was limited. Some of the bacterial genera appeared to show a sex-based selectivity, as they were only present in one sex. For example, *Novosphingobium* was exclusively found in females, whereas *Salinicoccus* was only detected in males, both with low abundance. At the species level, only *Pseudomonas aeruginosa*, *Shewanella algae* and *Staphylococcus xylosus* were identified, all of which were detected in both fully engorged females and males.

**Table 2:** The relative abundances of bacteria at the genus level in *Melophagus ovinus.* “Other” indicates the sum of relative genus abundance for the genera excluding the top 11

Sample	Relative abundance (%)											
	Bar	Ar	Wo	En	Ac	Ha	Sh	St	Bac	No	Sa	Other
Female	40.56	55.69	3.34	0.28	0.084	0.013	0.008	0.008	0.003	0.002	0	0.012
Male	59.91	38.14	1.67	0.2	0.055	0.006	0.008	0.002	0.003	0	0.003	0.003

*Abbreviations: Bar Bartonella*, *Ar Arsenophonus*, *Wo Wolbachia*, *En Enterobacter*, *Ac Acinetobacter*, *Ha Halomonas*, *Sh Shewanella*, *St Staphylococcus*, *Bac Bacillus*, *No Novosphingobium*, *Sa Salinicoccus*

## Discussion

Microbial community structures in the midgut of fully engorged female and male *M. ovinus* were investigated using Illumina HiSeq high-throughput sequencing. Our results showed certain bacterial genera that had already been reported in previous studies of *M. ovinus*, such as *Bartonella* and *Acinetobacter*, and we also detected bacterial genera that have not yet been reported in *M. ovinus*, including *Arsenophonus*, *Wolbachia*, *Enterobacter*, *Halomonas*, *Shewanella*, *Bacillus* and *Staphylococcus*.

*Bartonella* is a Gram-negative, haemotropic, fastidious, aerobic bacterium capable of intracellular parasitism [26]. Pathogenic *Bartonella* can cause many diseases, including Salonica fever, cat-scratch fever, and Carrin’s disease. *Bartonella* has a wide range of parasitic hosts and can infect humans and a variety of other vertebrates [27]. The main vectors are blood-sucking parasitic arthropods, such as lice, chiggers [28] and ticks [29], and mammals are the main reservoir hosts. When *Bartonella* infect a host, the bacteria first proliferate in endothelial cells and are later released into the blood, thereby infecting erythrocytes. When a haematophagous arthropod feeds on an infected host, *Bartonella* bacteria enter the vector, which then bites a healthy host, transferring the bacteria to the new host via the saliva and causing disease [28]. In the present study, we found a higher abundance of *Bartonella* in the midgut of *M. ovinus* than other relatively common bacterial genera, with males (59.91%) showing higher levels than females (40.56%). This finding suggests that *M. ovinus* can harbour a mass of *Bartonella*, which can adapt well to the midgut of this insect.

*Arsenophonus*, an intracellular symbiotic bacterium with a wide range of hosts and a high degree of biological diversity [30], was also present in the *M. ovinus* midgut, with females (55.69%) having higher levels than males (38.14%). Duron et al. [31] examined *Arsenophonus* 16S rDNA in 136 species of wild arthropods and found six species to be infected. *Arsenophonus*, which is extensively and non-specifically distributed in different tissues and organs of host insects, plays an important role in killing male hosts, allowing female hosts to obtain more resources and reduce adverse impacts due to inbreeding within the population. Gherna et al. [32] discovered that when *Nasonia vitripennis* become infected with *Arsenophonus*, approximately 80% of male progeny die; this phenomenon can result in a preference for female hosts. In previous studies, *Arsenophonus* was demonstrated to provide vitamins and other nutrients to host insects [33]. However, further research is required to determine whether the high abundance of *Arsenophonus* in the midgut of *M. ovinus* is related to nutrient provision.

*Wolbachia* is a cytoplasmically inherited intracellular symbiotic bacterium that can cause changes in a host’s reproductive behaviour [34, 35]. *Wolbachia* naturally infects a wide range of hosts, including nematodes [36], crustaceans and mites [37, 38] and dipteral insects [39]. According to a previous report, 60% of terrestrial insect species are infected with *Wolbachia* [40], which exists mainly in the ovaries and spermaries of insect hosts. In the ovary, *Wolbachia* mainly affects trophoblast cells, which participate in oogenesis as nutrient carriers. *Wolbachia* can induce reproductive manipulation of cytoplasmic incompatibility and parthenogenesis in the process of transport, thus regulating reproductive behaviour [41]. These bacteria are also found in other tissues, for instance, in the head, chest, midgut, Malpighian tubules and blood lymph tissues of adult *Drosophila* [42]. In the present investigation, we identified *Wolbachia* in the midgut of *M. ovinus*, with females (3.34%) exhibiting higher levels than males (1.67%).

*Acinetobacter* is a Gram-negative, obligate aerobic coccobacillus present in the normal flora of humans and animals and is widely distributed in natural environments (i.e. water, soil, mud, living organisms and vegetables) [43–46]. An opportunistic pathogenic bacterium, *Acinetobacter* frequently causes various types of infections, especially in immunocompromised individuals and in patients in intensive health care units. *Acinetobacter* has been detected in ticks and many types of arthropods. Kumsa et al. [47] performed molecular detection of *Acinetobacter* species in lice and *M. ovinus* from domestic animals in Oromia Regional State and found *Acinetobacter lwoffii* and a new *Acinetobacter* spp. (*Acinetobacter* sp*. G13*) in *M. ovinus* from sheep. In the present study, *Acinetobacter* was found in high abundance in the midgut of *M. ovinus*, though we could not identify the species.

*Rickettsia* is an obligate, intracellular, parasitic bacterium that is transmitted mainly through arthropods. In previous studies, *Rickettsia* was detected in *M. ovinus* collected in Hungary, with a prevalence of 1.67% (1/60) [13]. Recently, high prevalence rates (12.63%, 12/95) for *R*. *raoultii* and *R*. *slovaca* were reported for *M. ovinus* from Taklimakan Desert in China [8]. However, two studies reported a failure to detect *Rickettsia* spp*.* in *M. ovinus* collected in Ethiopia and the Czech Republic [18, 47]. Using next-generation sequencing, we similarly were unable to detect *Rickettsia* in two groups of *M. ovinus* collected from Jiuquan in Gansu Province, China. This finding suggests that the capacity of *M. ovinus* to carry *Rickettsia* may depend on the geographical region.

## Conclusions

*Bartonella*, *Arsenophonus* and *Wolbachia* were dominant bacterial genera in the midgut of fully engorged female and male *M. ovinus*. Although detected, *Enterobacter*, *Acinetobacter*, *Halomonas*, *Shewanella*, *Bacillus* and *Staphylococcus* showed low abundances. Importantly, this is the first report of *Arsenophonus*, *Wolbachia*, *Enterobacter*, *Halomonas*, *Shewanella*, *Bacillus* and *Staphylococcus* in the midgut of *M. ovinus*.

## Abbreviations

OTUs: Operational taxonomic units
